# Current News

**Published:** 1905-03

**Authors:** 


### Current News.

### OFFICERS OF THE LEWIS AND CLARK DENTAL CONGRESS.

### GENERAL COMMITTEE.

Norris R. Cox, D.D.S., Chairman, Abington Building, Portland.

Arthur W. Chance, D.D.S., M.D., Secretary, 809–810 Dekum Building, Portland.

### OREGON.

*State Dental Association.*—E. G. Clark, Portland; W. A. Cumming, Portland; Norris R. Cox, Portland; Walter F. Lewis, Portland; G. H. Nottage, Portland; Arthur W. Chance, Portland.

*Stomatological Club.*—S. J. Barber, Portland; J. B. Keefer, Portland; Geo. A. Marshall, Portland.

### CALIFORNIA.

*State Dental Association.*—H. B. Carlton, San Francisco; Jas. G. Sharp, San Francisco; C. E. Post, San Francisco.

*Southern California Dental Association.*—L. E. Ford, Los Angeles; Ray D. Robinson, Los Angeles; G. A. White, Santa Barbara. Los Angeles Alumni, H. D. Regua, Los Angeles.

*San Francisco Dental Association.*—A. M. Flood, San Francisco; J. S. Marshall, Presidio; F. C. Pague, San Francisco.

*Alameda County Dental Society.*—J. Loran Pease, Oakland; H. G. Chappel, Oakland; John S. Engs, Oakland.

### BRITISH COLUMBIA.

*British Columbia Dental Association.*—R. Ford Verrinder, Victoria; K. C. MacDonald, Grand Forks; Richard Nash, Victoria.

### WASHINGTON.

*State Dental Association.*—C. S. Irwin, Vancouver; E. S. Barnes, Seattle; C. A. Custer, Seattle.

*Seattle Dental Club.*—E. B. Edgers, Seattle; J. W. Ball, Seattle; Gregor McGregor, Seattle.

*Tacoma Dental Club.*—B. S. Scott, Tacoma; J. M. Myer, Tacoma; R. S. Williams, Tacoma.

*Spokane Dental Club.*—A. Stark Oliver, Spokane; Chas. C. Mann, Spokane; Jos. W. Downing, Spokane.

IDAHO.

*State Dental Association.*—J. B. Burns, Payette; J. H. Lewis, Nez Perce; E. H. Maberly, Boise.

MONTANA.

*State Dental Association.*—J. B. Keenan, Butte; R. M. Leslie, Great Falls; A. C. Sandberg, Butte; T. M. Hampton, Helena; J. D. Sutphen, Helena; Joseph Oettinger, Missoula.

UTAH.

*State Dental Association.*—W. G. Dalrymple, Ogden; W. Lean Ellerbeck, Salt Lake; E. A. Tripp, Salt Lake.

NEVADA.

J. C. Hennessy, Reno.

*Executive Committee.*—Norris R. Cox, Portland, Chairman; C. S. Irwin, Vancouver, Wash.; Arthur W. Chance, Portland, Secretary; Jean Cline, Portland; E. G. Clark, Portland.

*Committee on Essays.*—S. J. Barber, Portland, Chairman; C. L. Goddard, San Francisco; B. F. Eschelman, Tacoma.

*Committee on Clinics.*—G. H. Nottage, Portland, Chairman; J. M. Meyer, Tacoma, Wash.; C. E. Post, San Francisco; A. Stark Oliver, Spokane, Wash.; Claude W. Gates, Salt Lake, Utah; J. H. Holmes, New Westminster, B. C.; A. W. Cate, Boise, Idaho; W. H. Barth, Great Falls, Mont.; F. I. Shaw, Seattle, Wash.

*Committee on Exhibits and Manufacturing Clinics.*—W. F. Lewis, Portland, Chairman; Woodard, Clarke & Co., Portland; Archer & Schanz Company, Portland; John Welch Dental Depot, Portland; Oregon Dental Supply Co., Portland.

---

### AMERICAN DENTAL SOCIETY OF EUROPE.

THE next annual meeting of the American Dental Society of Europe will take place at Geneva, Switzerland, April 21 to 24, next. A cordial invitation is extended to members of the profession.

CHARLES J. MONK,
*Honorary Secretary.*

